# *Leptospira interrogans* insoluble fraction as a potential antigen source for lateral flow immunochromatography

**DOI:** 10.1590/0074-02760220265

**Published:** 2023-05-12

**Authors:** Hevandro de Souza Campos, Edimilson Domingos da Silva, Gerson Silva de Lima, Rafael de Oliveira Resende, Patricia Burth

**Affiliations:** 1 Universidade Federal Fluminense, Departamento de Biologia Celular e Molecular, Niterói, RJ, Brasil; 2 Universidade Federal Fluminense, Programa de Pós-Graduação em Biotecnologia, Niterói, RJ, Brasil; 3 Fundação Oswaldo Cruz-Fiocruz, Bio-Manguinhos, Rio de Janeiro, RJ, Brasil; 4 Fundação Oswaldo Cruz-Fiocruz, Instituto Oswaldo Cruz, Instituto Nacional de Ciência e Tecnologia em Neuroimunomodulação, Laboratório de Pesquisas sobre o Timo, Rio de Janeiro, RJ, Brasil; 5 Universidade Federal de Uberlândia, Instituto de Ciências Biomédicas, Uberlândia, MG, Brasil

**Keywords:** leptospirosis, lateral flow, immunochromatography, diagnosis

## Abstract

**BACKGROUND:**

Leptospirosis is an emerging zoonosis that affects humans and animals. Immunochromatography rapid test is widely used for early diagnosis of leptospirosis, but with low sensitivity and specificity.

**OBJECTIVES:**

To evaluate *Leptospira interrogans* insoluble fraction as a potential antigen source for lateral flow immunochromatography.

**METHODS:**

Insoluble fraction derived from the crude bacterial extract was obtained by serial centrifugation. The polypeptide profile was determined using sodium dodecyl sulfate-polyacrylamide gel electrophoresis (SDS-PAGE). Immune reactivity of this fraction was assessed by Western Blotting and lateral flow immunochromatography (LFI). It was tested 160 microagglutination test (MAT)-positive sera from patients in the acute phase, 100 MAT-negative sera from patients with acute febrile illness, and 45 patients with other infectious diseases.

**FINDINGS:**

There was a predominance of low molecular mass-polypeptide bands, ranging from 2 to 37 kDa. The antibody reactivity of theses polypeptides was found to range from 13-50%, especially between 10 and 38 kDa. Among MAT-positive sera of patients with leptospirosis in the acute phase, 97% were also positive in LFI, indicating high sensitivity. Among MAT-negative sera, all were negative in LFI, indicating high specificity. Only 2% of cross-reactivity was detected.

**CONCLUSION:**

The insoluble fraction can be a valuable antigen source for development of point-of-care diagnosis test for leptospirosis.

Leptospirosis, in humans, is acquired through contact with animals, which are reservoirs for *Leptospira*, or an environment contaminated with their urine. The infection can be identified by general clinical manifestations, such as fever, headache, and myalgia in mild cases, which may progress to renal failure, jaundice, and pulmonary haemorrhage.^1^ ^,^ ^2^ In the acute phase, the bacterial lysis releases the glycolipoprotein fraction (GLP), one of the most important *Leptospira* endotoxins. Although this fraction is involved in the main pathophysiological events in leptospirosis, however, it had not been identified until 1995, when our team described the association of GLP fraction and Na^+^K^+^ ATPase, including manifestation in the kidney, liver, and lung.^3^ ^,^ ^4^

Due to unspecific clinical symptoms and the prevalence of other bacterial infections, particularly in endemic areas, it is important to provide an accurate diagnosis.^5^ The microagglutination test (MAT) is the gold standard and it is based on the agglutination of immune components that reacts against *Leptospira* strains with antibody titres visually determined by a dark-field microscope.^6^ The enzyme-linked immunosorbent assay (ELISA) can also detect specific IgM or IgG antibodies to *Leptospira* antigens with higher sensitivity, but reasonably low specificity.^7^ It is often carried out using *L. interrogans* or *L. biflexa* antigens and results can be obtained in a few hours.^2^ ^,^ ^8^ Both procedures are difficult to execute, requiring specialised professionals and equipped laboratories.

For point-of-care diagnosis, immunochromatography-based tests detect IgM and IgG and provide an additional advantage by not requiring robust equipment.^9^ For leptospirosis, in addition to the lateral flow immunoassay (LFI), a double-path platform test (DPP) can also detect specific antibodies in few minutes.^10^ ^,^ ^11^ Both tests are based on a cassette containing a 0.05-12 μm pore size nitrocellulose membrane associated to other polymers and conjugated molecules.^12^ The DPP assay for leptospirosis was developed by Chembio Diagnostic Systems (Medford, New York, USA) and it is manufactured and distributed in Brazil by Bio-Manguinhos/Fiocruz through a technology transfer agreement with the Brazilian Ministry of Health. In this test, high concentrations of recombinant *Leptospira* immunoglobulin-like proteins (rLig) were used as an antigen source. It is based on IgM and IgG detection, highly sensitive for the acute phase compared to ELISA, but with 73% sensitivity for mild disease. Within seven days of disease onset, sensitivity drops to 77% and 60% for severe and mild diseases, respectively.^10^ Additionally, Smits et al.^13^ described an LFI assay for the detection of *Leptospira-*specific IgM antibodies in human sera, using a broadly reactive *Leptospira* antigen recognised by anti-human IgM/colloidal gold, with 85% and 94% sensitivity and specificity, respectively. Despite using recombinant protein and total bacterial extract in a solid phase, no other antigen fraction has been assessed as a potential tool for a rapid diagnosis platform. Thus, we aimed to evaluate an insoluble fraction derived from the *Leptospira* crude extract as an alternative antigen source on LFI for detecting specific IgM antibodies.

## SUBJECTS AND METHODS

*Subjects* - The study was approved by the local ethics committee, in affiliation with the Brazilian Ministry of Health (protocol 24215713.7.0000.00.40). Sera (n = 305) were characterised by Instituto Gonçalo Muniz/Fiocruz and Bio-Manguinhos/Fiocruz, and included in groups 1, 2, 3, and 4, based on their immune profile, as described in Table I. Additionally, eight sera (seven positive and one negative) obtained from a serological panel previously characterised by MAT and DPP for leptospirosis were used for screening membrane test.

**TABLE I:** Immunological profile of sera samples

Group	n	Description
1	100	Patients with leptospirosis in acute phase with positive diagnosis, determined by microagglutination test (MAT)
2	100	Patients with acute febrile illness from endemic area for leptospirosis with negative diagnosis, determined by MAT
3	60	Patients hospitalised with leptospirosis in convalescent phase, with positive diagnosis, determined by MAT
4	45	Patients with hepatitis C (HCV) (10), hepatitis B (HBV) (10), HIV (10) and syphilis (15) from a sera panel

*Leptospira interrogans fraction* - *L. interrogans* (serovar Canicola) strain was kindly provided by the National Reference Laboratory for Leptospirosis, Instituto Oswaldo Cruz (IOC/Fiocruz), being maintained and expanded as described elsewhere.^14^ For bacterial lysis, the suspension was kept in the ice bath and submitted to sonication, three cycles of 20 s, 25 s intervals, 22% amplitude. The lysate was centrifuged at 14,000 x *g* for 30 min and the insoluble fraction (IN) was stored at -80ºC until the tests were carried out. The protein content was determined by the BCA method (ThermoFisher Scientific*,* Rockford, USA) in polystyrene microplates (Nunc, ThermoFisher), according to the manufacturer’s instructions.

*SDS-PAGE* - The polypeptide profile of the IN fraction was determined by polyacrylamide gel electrophoresis in 12% sodium dodecyl sulfate-polyacrylamide gel electrophoresis (SDS-PAGE) (NuPAGE, ThermoFisher) with 7 µg protein/well in sample buffer (10% glycerol, 0.6% SDS, 0.25 M Tris-HCL, pH 6,8), added to 10% β-mercaptoethanol, 0.04% bromophenol blue and 0.25 mM dithiothreitol (DTT), denatured at 95ºC for 5 min and centrifuged at 1,000 x g for 1 min. Polypeptide molecular masses were calculated by Gel Doc XR+, version 4.1 (Bio-Rad, Hercules, USA), with SeeBlue Plus2 (ThermoFisher*)* as reference.

*Western Blotting* - Western Blotting was carried out to assess the immunological reactivity of *Leptospira*-positive sera and control individuals against IN.^15^ Samples were prepared for SDS-PAGE, and polypeptides were transferred to a nitrocellulose membrane and blocked with 2% skimmed milk (Sigma). Sera were diluted in phosphate-buffered saline (PBS)-1% Tween 20 (Sigma) (PBS-T) added of 0.1% skimmed milk and loaded onto the membrane, incubated by 30 min, at 37ºC, under slow agitation. Subsequently, the membrane was washed three times with TBS-T, under moderate agitation, at 37ºC, for 5 min, followed by anti-μ/HRP antibody (Bio-Manguinhos/Fiocruz). The reaction was developed with 0.2% 3,3’-diaminobenzidine (DAB) and 0.01% H_2_O_2_ in PBS. Polypeptide molecular masses were calculated as previously described.

*Membrane screening test* - DPP Leptospirosis, DPP Syphilis, and HIV1/2 Rapid Test diagnosis kits (Bio-Manguinhos/Fiocruz) were used as a model for LFI. The assay was conducted according to the manufacturer’s instructions. All steps, from cassette assembly to production of the running buffer and colloidal gold-labelled human anti-IgM conjugate (anti-IgM/gold) were conducted according to internal documents.

Sample printing and cassette assembling were performed with fixed values for filling rate (8 µL/s), IN fraction volume (10 µL), syringe volume (25 µL), and membrane length (190 mm). Three pore size conditions were used, as follows: 3 µm, 5 µm, and 10 µm. Both IN and anti-IgM/colloidal gold were printed by the IsoFlow Flatbed Dispenser (Imagene Technology, Hanover, USA). Initially, seven homogeneously pooled sera (n = 7) *Leptospira*-positive and one control serum (*Leptospira*-negative) from a serological panel were tested. Once the best condition was defined, serum samples were increased as described in details in Table I.

*LFI assay* - Small and large-scale LFI assays, as well as interpretation of results, were performed according to standard protocols for rapid test manufactured by Bio-Manguinhos/Fiocruz. To perform the text, 5 μL of each serum sample was applied to the appropriate well in the cassette, followed by the addition of three drops of the running buffer. After 10 min, the result was visually interpreted in the designated region of the cassette for line visualisation. A positive test result was indicated by the appearance of a pink/purple line in both T (test) and C (control) tags, while a negative result was indicated by the reactivity of only the C line. An invalid result was considered when the line C was not reactive.

*Statistical parameters* - All statistical analysis were performed by Graphpad Prism 6.0 (GraphPad). Sensitivity, specificity, and positive/negative predictive values of the LFI test using IN fraction were determined using MAT as golden standard. Differences between the positive and negative sera for LFI was evaluated by the χ^2^ method or Fisher’s Exact Test, when applicable. Results were significant at a significance level of p < 0.05.

## RESULTS

**Fig. 1:**
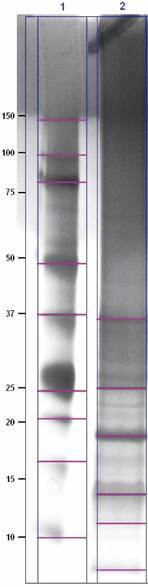
sodium dodecyl sulfate-polyacrylamide gel electrophoresis (SDS-PAGE) polypeptide profile of *Leptospira interrogans* insoluble fraction (IN) (Lane 2) and molecular mass standard (Lane 1), with respective values; pink lines indicate the main polypeptide bands; silver staining.

**Fig. 2:**
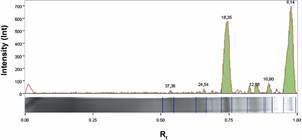
signal intensity of polypeptide bands from *Leptospira interrogans* insoluble fraction (IN). Relative mobility (Rf) is represented in association with molecular mass values and intensity.

*Immune reactivity to polypeptide bands* - A broad spectrum of low molecular mass polypeptides was found IN fraction, as shown in SDS-PAGE (Fig. 1). In total, 8, 11, 13, 18, 25, and 37 kDa bands were detected with 700, 100, 50, 600, 10, and 10 i relative intensity values, respectively (Fig. 2).

**Fig. 3:**
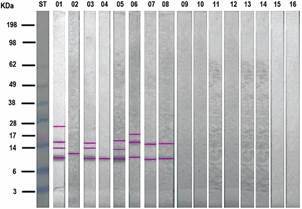
Western Blotting IgM reactivity profile of leptospirosis positive (1-8) and a negative serum against *Leptospira interrogans* insoluble fraction (IN). ST: molecular mass standard; pink lines indicate the main polypeptide bands.

**Fig. 4:**
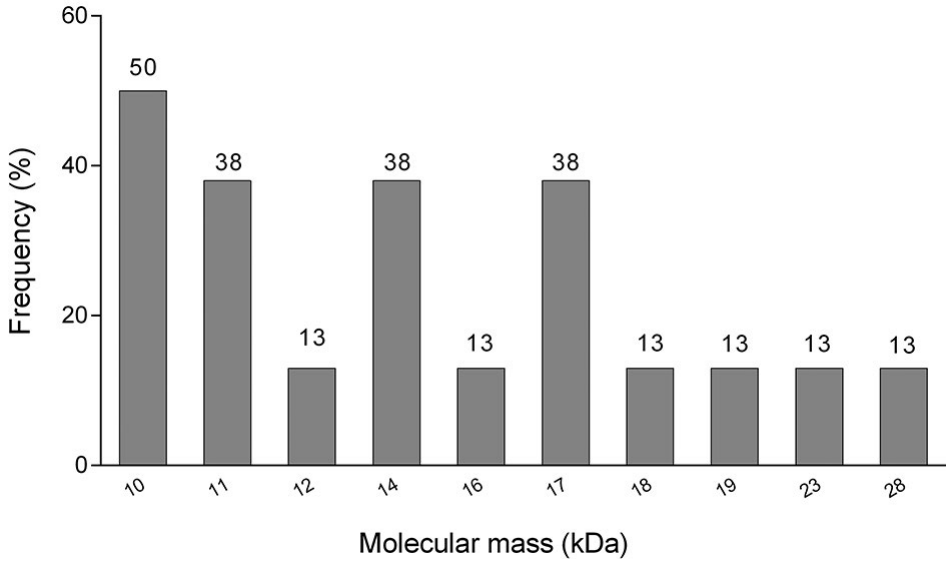
polypeptides recognised by microagglutination test (MAT)-positive sera against *Leptospira interrogans* insoluble fraction (IN) in Western Blotting.

The Western Blotting assay revealed a consistent recognition profile of the polypeptide bands in sera with high antibody titres. In the IN fraction (Fig. 3), there was a clear immunodominance towards low molecular mass polypeptides, particularly the 10 and 17 kDa bands, although other bands ranging from 11 to 28 kDa were also detected. Notably, over 50% of the sera recognised the 10 kDa band, while 38% of the sera the 11, 14 and 17 kDa bands and the others were recognised by 13% of the sera (Fig. 4).

**Fig. 5:**
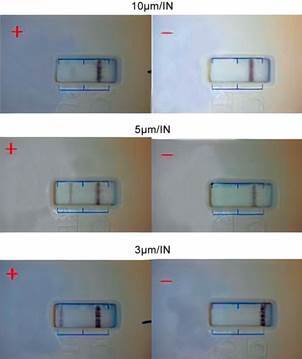
representative image of cassettes on nitrocellulose membrane test for lateral flow immunochromatography (LFI) using 3 µm, 5 µm, and 10 µm pore sizes loaded with *Leptospira interrogans* insoluble fraction (IN) associated with positive (+, n = 7, pooled) and negative (-, n = 1) sera.

*LFI assay* - In parallel, three nitrocellulose membranes were assembled to the cassette and assay were developed at the same conditions, using positive and negative control sera. Control line (C) was present in all membranes although prominent in 3 µm (Fig. 5). Test line (T) was present when using positive sera and absent in negative with better resolution in 3 µm, which has been chosen as the standard. After determining the optimal conditions for membrane and running buffer (Table II), the IN fraction was printed in a 3 µm nitrocellulose membrane and devices were assembled, constituting a lateral flow (LFI) platform for IgM detection. Positivity rate was visually labelled high (+++), medium (++), and weak (+). The negative result (-) was described by the absence of the T test line. In groups 2, 3, and 4, that include MAT-negative patients, convalescent phase, and other diseases, respectively, only 13 sera were positive to IN fraction, although poorly. Among the *Leptospira*-positive sera (group 1), 97 (97%) were also positive to IN fraction, and only 3 sera (3%) had a negative result (p < 0.0001), indicating considerable importance of using this fraction for LFI. Among the acute phase negative sera (group 2), the majority (97%, p < 0.0001) reacted similarly to LFI loaded with IN fraction (Table III). The positive and negative predictive values reached 97% [95% confidence interval (CI) = 91% - 99%] for all parameters. For cross-reactivity assessment, when positive sera to other infectious diseases were tested, a serum (2%) of a patient with syphilis was also positive for LFI.

**TABLE II:** Analytical profile of lateral flow immunochromatography (LFI) using insoluble fraction (IN) obtained from *Leptospira interrogans* lysate

Parameter	Value	Unit
Membrane length	190	mm
Strip length	5	mm
Loaded volume/membrane	15	µL
Loaded volume/strip	0.39	µL
Protein concentration/membrane	143.97	µg/mL
Protein content/strip	0.057	µg

**TABLE III:**
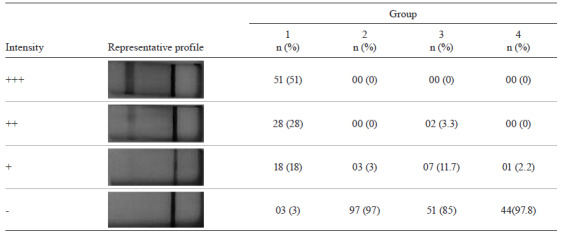
Immunological profile of sera from groups 1, 2, 3, and 4 against the insoluble fraction (IN) derived from *Leptospira interrogans* lysate, determined by lateral flow immunochromatography (LFI)

## DISCUSSION

Our study found that an insoluble fraction, obtained by serial centrifugations of *L. interrogans* extract, can be used in an immunochromatography assay for the diagnosis of leptospirosis. However, one of the main challenges in our study was to obtaining a sufficient yield after cultivating *Leptospira* bacterial mass in *Leptospira* Enrichment EMJH culture medium, which is known to have a low bacterial growth rate, as also described by others.^16^

Bacterial proteins constitute the main antigen basis for recognition by the immune system and this recognition pattern plays a crucial role in disease diagnosis. In SDS-PAGE, we observed prominent polypeptide bands at 8, 11, 13, 18, 25 and 37 kDa, especially from 11 to 25 kDa, which were also detected by previous studies.^17^ ^,^ ^18^

For LFI screening, it has been tested different pore sizes of nitrocellulose membranes using sera from a standard panel. To date, no study has used any protein fraction isolated from *Leptospira* as a solid phase in LFI. In addition to obtaining an insoluble fraction, our group has suggested an analytical profile of these proteins associated to immune reactivity to provide an accurate diagnosis test. Posthuma-Trumpie et al.^12^ have reported that membranes screening is crucial to ensure proper flow of immune and chemical components through the pores. Since IFL results rely on visual interpretation, the lines revealed by antigen-antibody binding must be clear and well-defined. Based on these factors, three membranes were assessed, and we found that 3 µm-pore size membrane yielded the best results.

Furthermore, 305 sera were tested, including those from patients positive to other pathogens. This step is important for detecting potential cross-reactivity between *Leptospira* and other in the same endemic area, as reported by Smits et al.,^11^ which used positive sera for HIV, hepatitis A, hepatitis B, syphilis, malaria, toxoplasmosis, meningitis, meningococcal meningitis, reporting cross-reactivity with later. Amran et al.^19^ also described cross-reactivity with dengue, syphilis, and typhoid. In the platform used in our study, although evidence of this cross-reactivity has also been found indicating a potential homology with *Treponema pallidum* components, these findings must be explored to clear the molecular mechanisms involved in this immune reactivity.

DPP immunochromatography assay has been used for leptospirosis diagnosis although low sensitivity has been reported as a limitation. A study reported 73% sensitivity with samples from patients with leptospirosis who have been submitted to DPP with recombinant Lig proteins.^10^ However, we showed that IN fraction derived from *Leptospira* lysate on a rapid test platform can increase sensitivity to 93%.

The discordance rate in groups 1 and 2 was only 3%. The high reactivity found in acute samples compared to convalescent samples was an expected result, since the conjugate used in the IFL was specific for IgM detection. Our study evaluated sensitivity, specificity, and predictive values, with consistent results. The high sensitivity (97%) indicates a wide range of reactivity, and the specificity is also expressive, indicating few false positive samples. Our results demonstrate an advantage in relation to others that achieved < 95% specificity and 73% sensitivity in patients with mild disease.^10^ Smits et al. obtained 86% sensitivity and 94% specificity while Alamuri et al., using a recombinant surface antigen Lsa27, revealed 90% and 91% sensitivity and specificity, respectively.^13^ ^,^ ^19^ ^,^ ^20^ ^,^ ^21^ ^,^ ^22^ In other study, Alia et al. reported 40% sensitivity and 89% specificity, with immunochromatography tests carried out with samples previously characterised by MAT, while others revealed 91% sensitivity and 83% specificity.^23^ ^,^ ^24^ ^,^ ^25^ ^,^ ^26^ Dinhuzen et al.^27^ described 40% sensitivity and 89% specificity using the same test. Comparative pattern among these studies is presented in Table IV.

**TABLE IV:** Sensitivity and specitivity values for leptospirosis diagnosis achieved in the current study (Campos et al.) and other reports

Reference	Sensitivity (%)	Specificity (%)	Test
Campos et al.	97	97	LFI
10	95	73	DPP
19	73	90	LFI
13	86	94	LFI
20	92	96	Immunoblot IgM
21	72	62	PCR
22	90	91	Latex aglutination test
23	40	89	LFI
23	91	83	LFI
27	40	89	LFI

DPP: double-path platform test; LFI: lateral flow immunochromatography; PCR: polymerase chain reaction.

Overall, despite of the small sample size, our study showed that the IN fraction is a useful antigen source for LFI, as it achieved high specificity and sensitivity (97%). These findings suggest that this fraction can be a reliable alternative for developing a robust diagnostic platform for leptospirosis, which is critical for addressing public health concerns.
